# Mesh-Tissue Integration of Platelet-Rich Plasma–Decellularized Amnion Scaffold–Polypropylene Mesh Sandwiches Implanted in the Vesicovaginal Spaces of Hypoestrogenic Rabbit Models: Protocol for a Randomized Controlled Trial

**DOI:** 10.2196/37942

**Published:** 2022-08-09

**Authors:** Alfa Putri Meutia, Budi Iman Santoso, Andon Hestiantoro, Puspita Eka Wuyung, Joedo Prihartono, Arief Boediono, Suskhan Djusad, Amir Fauzi, Pribakti Budinurdjaja

**Affiliations:** 1 Doctoral Program in Medical Sciences Faculty of Medicine Universitas Indonesia Jakarta Indonesia; 2 Department of Obstetrics and Gynecology Faculty of Medicine Universitas Indonesia - Dr. Cipto Mangunkusumo National Referral Hospital Jakarta Indonesia; 3 Department of Pathology Anatomy Faculty of Medicine Universitas Indonesia - Dr. Cipto Mangunkusumo National Referral Hospital Jakarta Indonesia; 4 Department of Community Medicine Faculty of Medicine Universitas Indonesia - Dr. Cipto Mangunkusumo National Referral Hospital Jakarta Indonesia; 5 Department of Anatomy, Physiology and Pharmacology Institut Pertanian Bogor University Bogor Indonesia; 6 Division of Urogynecology and Reconstructive Surgery, Department of Obstetrics and Gynecology Faculty of Medicine Universitas Sriwijaya - Dr. Mohammad Hoesin General Hospital Palembang Indonesia; 7 Division of Urogynecology and Reconstructive Surgery, Department of Obstetrics Obstetrics and Gynecology Faculty of Medicine Universitas Lambung Mangkurat - Ulin General Hospital Kalimantan Selatan Indonesia

**Keywords:** pelvic organ prolapse, vaginal mesh, platelet-rich plasma

## Abstract

**Background:**

Mesh-augmented surgery with polypropylene meshes (PPMs) is often used in urogynecology and pelvic reconstructive surgery. However, the various complications that arise from its integration process have resulted in a decrease in the number of mesh-augmented surgeries performed worldwide. An approach to improving mesh-tissue integration is coating PPMs with anti-inflammatory and wound-healing molecules, such as platelet-rich plasma (PRP), which is a component of biotechnologies that are capable of accelerating wound healing. Estrogen is also known to have a beneficial effect on wound remodeling; therefore, a hypoestrogenic status may have negative implications for wound healing. The mechanism of how PRP plays a role in wound remodeling, especially among individuals in a hypoestrogenic state, has not been fully described until now.

**Objective:**

Our aim is to investigate the impact of applying PRP to PPMs in hypoestrogenic rabbit models.

**Methods:**

Our study will be a randomized controlled trial involving hypoestrogenic rabbit models. Samples were categorized into either the PRP group or the PPM group (1:1 ratio), with a minimum sample size of 16 in each arm, via simple random sampling. All samples were put into a hypoestrogenic state via bilateral oophorectomy. After confirming a decrease in estradiol level, the meshes were implanted in the vesicovaginal space. The samples were euthanized on the 14th, 28th, or 90th day of the surgery. The mesh-tissue integration process will be analyzed based on inflammatory parameters (inflammatory infiltrate, interleukin-17, and interleukin-1B expression); angiogenesis (CD31 expression); and collagen deposition, which will be assessed by using Masson trichrome staining.

**Results:**

Our study is in the protocol development stage. A preliminary study regarding its feasibility, including the feasibility of the preparation of hypoestrogenic rabbit models, mesh implantation in the rabbits’ vesicovaginal spaces, the PRP and amnion scaffold, started in February 2022. The results of our study are expected to be available by the end of 2022.

**Conclusions:**

Our randomized controlled trial is designed to provide high-quality evidence on the effect of applying a PRP-decellularized amnion scaffold to PPMs in the vesicovaginal spaces of hypoestrogenic rabbit models.

**International Registered Report Identifier (IRRID):**

PRR1-10.2196/37942

## Introduction

There are various management strategies for stress urinary incontinence and pelvic organ prolapse (POP), including mesh-augmented surgery using polypropylene meshes (PPMs). Sacrocolpopexy is an excellent alternative to apical repair that provides satisfactory results, with a level of effectiveness of 76% to 96% and a recurrence rate of 7.4% [1]. Although mesh-augmented surgery has high effectiveness and low recurrence rates, mesh-related complications, such as vaginal discharge, infection, chronic pain, adhesion, and extrusion/erosion, can harm women's quality of life. These adverse results from the implant can be related to abnormalities in the wound healing process [2].

Wound healing is a physiological reaction to tissue injuries and involves the following three overlapping phases: hemostasis/inflammation, proliferation, and remodeling. The inflammatory phase begins with the process of hemostasis and chemotaxis. Then, proinflammatory cytokines activate neutrophils, thereby limiting further damage and removing cellular debris and bacteria. Angiogenesis, re-epithelization, collagen formation, and injury contraction occur in proliferation. Any disruption to these phases leads to excessive wound healing or chronic wound formation. Unfortunately, most patients with POP are in a menopausal state, implying that hypoestrogenic conditions may also contribute to poor wound healing. Moreover, implantations adjacent to vaginal tissue, which is known to have typical flora, may also further disrupt the wound healing process [3,4].

Platelet-rich plasma (PRP) is known as a platelet concentrate with a 3 to 5 times higher than average platelet count that is obtained through the centrifugation of a blood sample. The potential therapeutic effect of PRP is based on its ability to improve tissue regeneration by releasing growth factors present in platelet α-granules [5,6], Considering its effects on the hemostasis, proliferation, and remodeling phases of wound healing, PRP could be used as a coating agent for PPMs in urogynecology and pelvic reconstructive surgeries. However, animal studies have been suggested to better ascertain a host's response to PRP-coated implants, especially in hypoestrogenic conditions.

This protocol was designed to investigate the changes in the mesh-tissue integration of PRP-decellularized amnion scaffold (DAS)–PPM sandwiches implanted in the vesicovaginal spaces of hypoestrogenic rabbit models.

## Methods

### Study Design and Population

**Figure 1:**
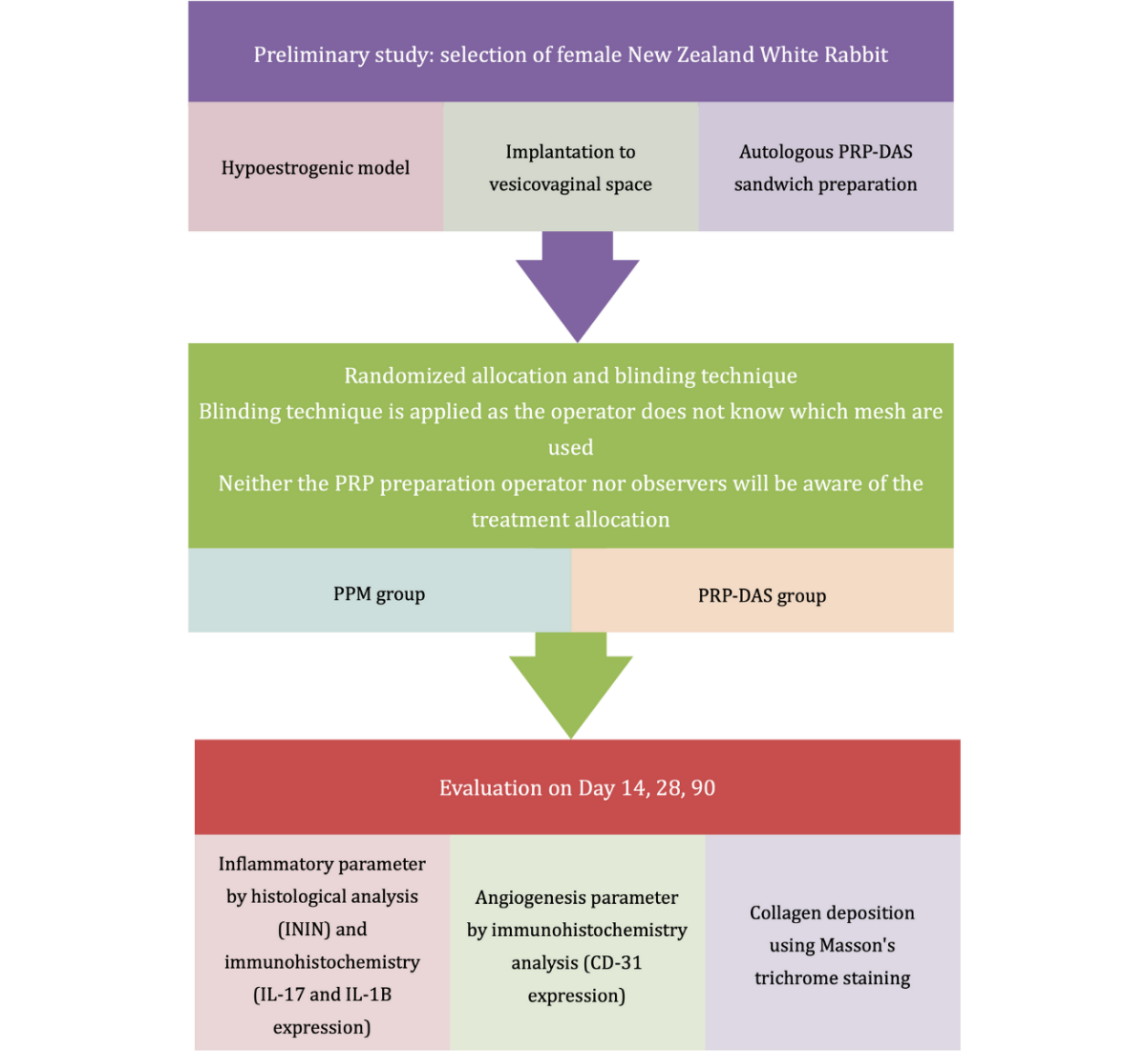
Study process flowchart. DAS: decellularized amnion scaffold; IL: interleukin; ININ: inflammatory infiltrate; PPM: polypropylene mesh; PRP: platelet-rich plasma.

Our study is a randomized controlled animal trial that aims to investigate the changes in the mesh-tissue integration of PRP-DAS–PPM sandwiches implanted in the vesicovaginal spaces of hypoestrogenic rabbit models. The study plan for the proposed research is shown in Figure 1.

### Ethics Approval

The approval for the protocol of our study was granted by Animal Research Ethical Committee, Faculty of Veterinary Medicine, Institut Pertanian Bogor University, in September 2021 (ethical clearance number: 208 - 2021 IPB), and data collection commenced in February 2022. The protocol was prepared according to the ARRIVE (Animal Research Reporting In Vivo Experiments) 2013 checklist for reporting an animal study.

### Hypoestrogenic Rabbit Model

A hypoestrogenic state was induced via bilateral oophorectomy. A 3-cm incision was made on the midline, and the identification of the uterus and mesovarium, followed by the ligation of ovaries using Vicryl 3.0 (Ethicon), was done. Estradiol levels before and after oophorectomy were measured via an enzyme-linked immunosorbent assay using the Estradiol Parameter Assay Kit (catalog number: KGE014; R&D systems). Blood samples were obtained from all rabbits by using a 23-gauge wing needle on the auricular vein and stored at room temperature for 30 minutes to allow for clotting. The samples were then centrifugated for 15 minutes at 1000 revolutions per minute. The serum was removed, pretreated, and assayed immediately. A hypoestrogenic state was confirmed when there was a ≥50% decrease in estradiol levels from baseline [7].

### Vesicovaginal Mesh Implantation

**Figure 2:**
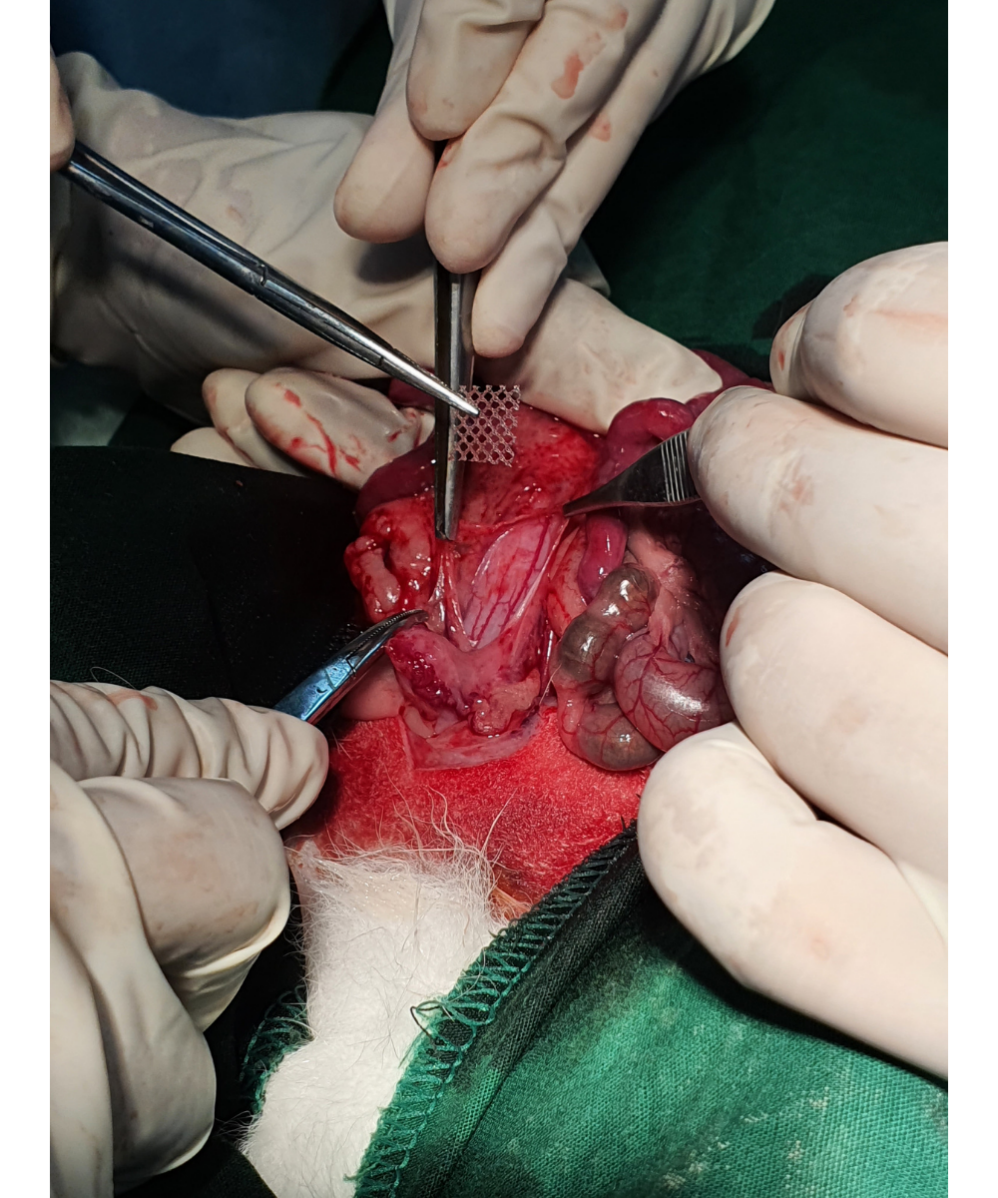
Image of the polypropylene mesh.

**Figure 3:**
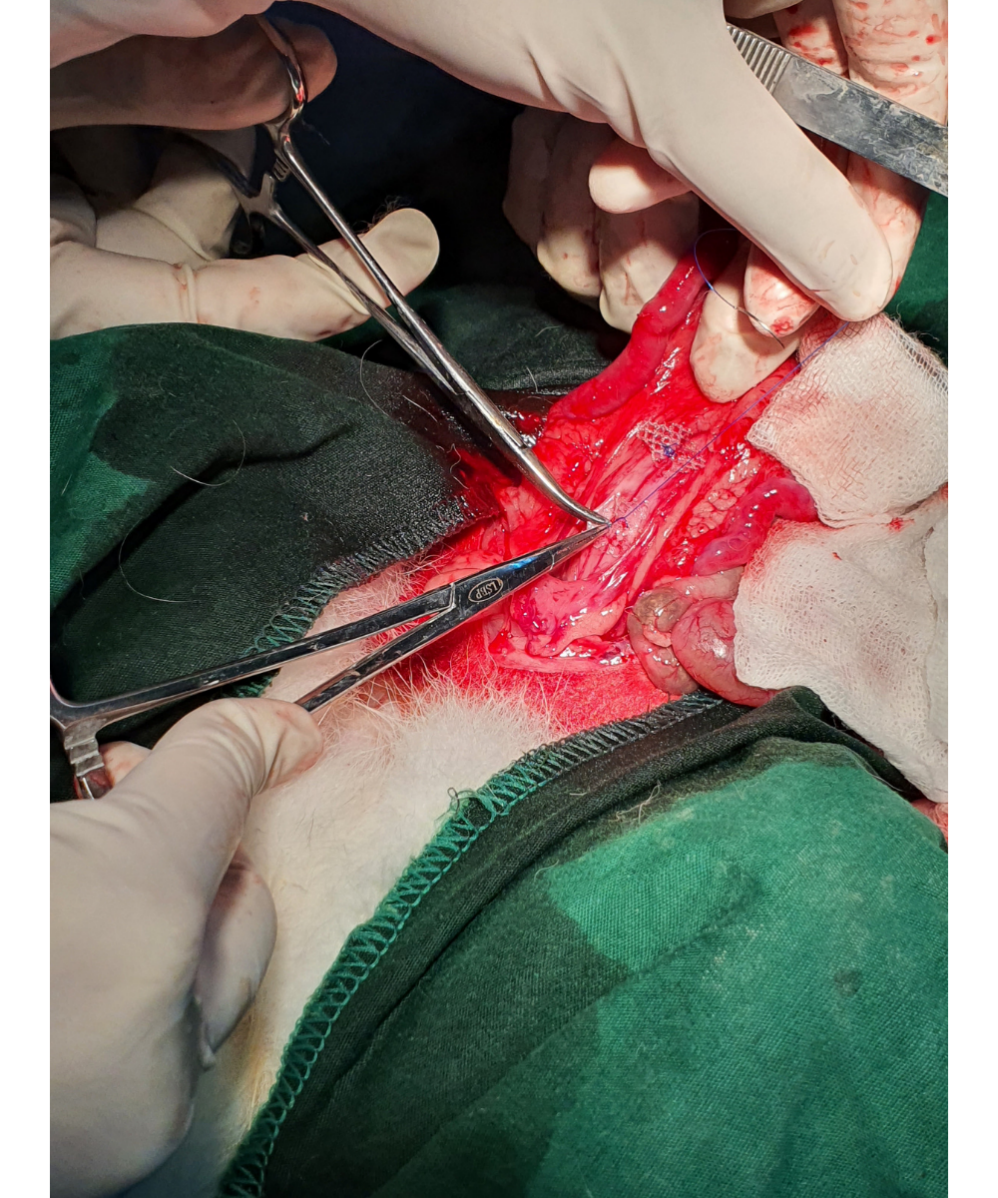
Application of the polypropylene mesh in the vesicovaginal space of a hypoestrogenic rabbit model.

The implantation of a PPM into the vesicovaginal wall was done by making a 3-cm incision on the midline of the lower abdominal wall, during which the bladder and uterus were identified. A further dissection was made toward the vesicovaginal space, and a 1×1-cm mesh was implanted on the anterior vaginal wall, as shown in Figure 2. A fixation suture using Prolene 3.0 (Ethicon) was made as a marker for tissue harvests in the future, as shown in Figure 3.

### PRP-DAS–PPM Sandwich

**Figure 4:**
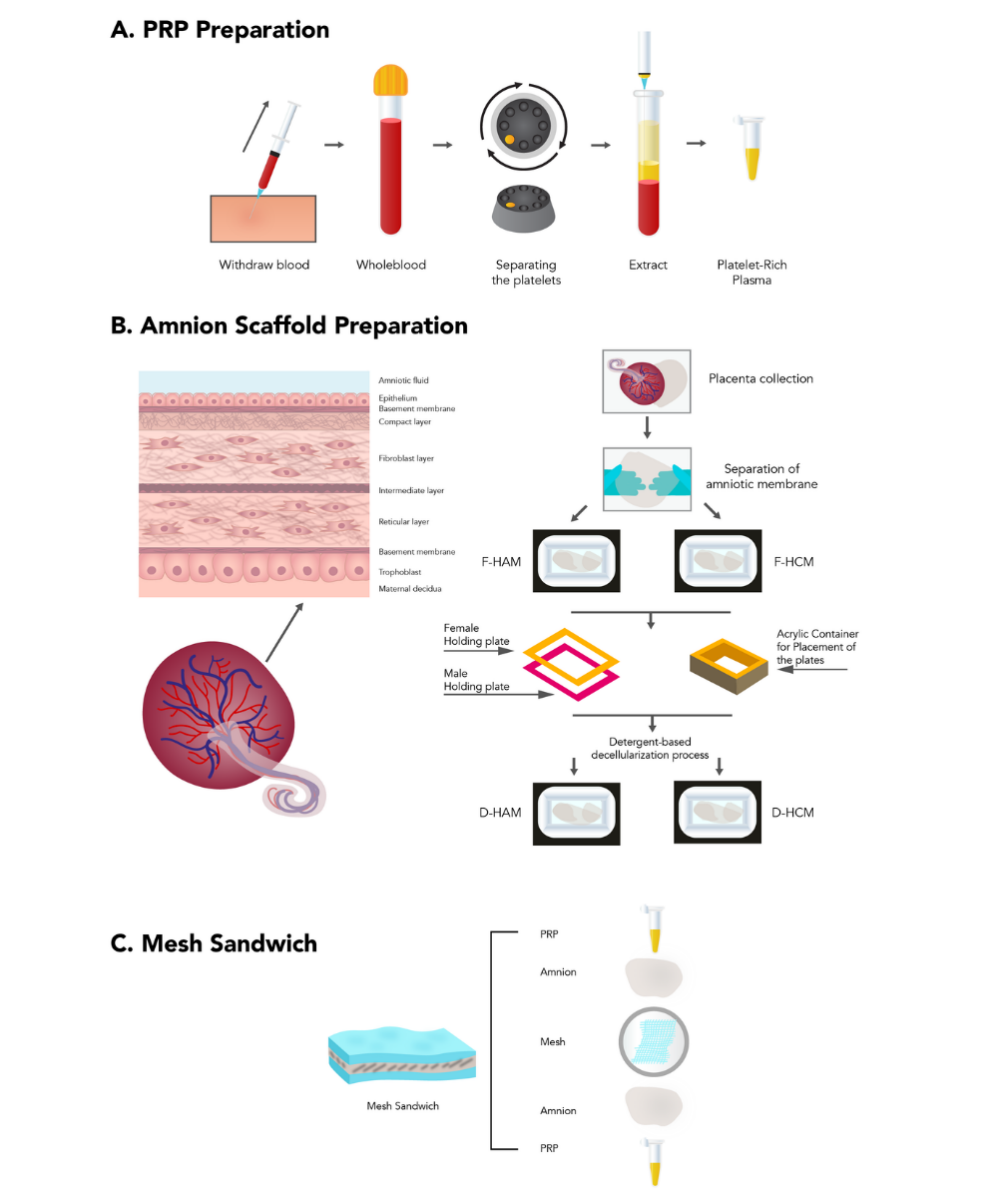
Proposed protocols for PRP-decellularized amnion scaffold–polypropylene mesh sandwich preparation. D-HAM: decellularized human amniotic membrane; D-HCM: decellularized human chorionic membrane; F-HAM: fresh human amniotic membrane. F-HCM: fresh human chorionic membrane; PRP: platelet-rich plasma.

Blood samples were taken from each subject by using a 23-gauge wing needle on the auricular vein. By using an autologous PRP preparation process that followed the standard protocol of our center, a platelet count was done before and after PRP preparation to ensure that the amount of platelets from each subject was standardized. A DAS was harvested from human placenta and collected in normal saline containing antibiotics (penicillin: 1000 IU/mL; streptomycin: 20 µg/mL, antifungal amphotericin B: 2.5 µg/mL). Under sterile conditions, the amnion will be separated from the chorion via blunt dissection under a laminar air hood. The separated amnion will be washed 2 to 3 times to remove all blood clots and blood. The amnion will be properly fixed on the cassettes of a bioreactor (19×14×0.3 cm). The amnion will be transferred to a 2% weight by volume sodium dodecyl sulphate solution, which will undergo gentle agitation on a shaker at 180 revolutions per minute for 12 hours at room temperature. After 12 hours, the amnion will be transferred into deionized distilled water and stored in a deep freezer overnight. The amnion will be thawed on the next morning at room temperature, and this cycle will be repeated 8 to 10 times. The processes of decellularization will be checked at every fifth cycle via hematoxylin-eosin staining and 4,6-diamnionidino-2-phenylindol staining to confirm cell removal. A 1×1-cm mesh will be covered by a PRP-DAS on both sides, forming a sandwich configuration, as shown in Figure 4.

### Histologic and Immunohistochemical Analyses

#### Histologic Analysis

An inflammatory infiltrate analysis will be carried out histologically by using samples that will be placed in a container filled with 10% formaldehyde for 48 hours at 4 °C, transferred to a 70% alcohol solution, and fixed in 10% formalin. Then, the samples will be immersed in liquid paraffin to make a paraffin block. After a 30-minute incubation period, the paraffin blocks will be heated at 60 °C for 5 to 10 minutes; incubated twice in xylene for 5 minutes; and then rehydrated for 5 minutes once in 100% ethanol, once in 90% ethanol, once in 70% ethanol, and twice in deionized water. The samples will be stained via the hematoxylin-eosin staining method and observed using a microscope at a magnification of ×10 to ×40.

Collagen deposition will be assessed by using Masson trichrome staining.

#### Immunohistochemistry Analysis

Inflammation and angiogenesis will be analyzed using immunohistochemistry. We will use tissue samples that will be made into paraffin blocks by using the same method mentioned in the *Histologic Analysis* section. The endogenous peroxidase in the paraffin blocks will be inactivated with 3% H_2_O_2_ for 10 minutes at room temperature, blocked with 10% normal goat serum for 30 minutes at room temperature, and then incubated with primary antibodies overnight at 4 °C. Subsequently, the samples will be incubated with biotinylated secondary antibodies for 30 minutes at room temperature. Antigen-antibody binding will be assessed by using a detection system tool, and immunohistochemical staining will be performed by using diaminobenzidine. Objective calculations will be performed by calculating the immunoreactive area, which will be multiplied by the intensity to calculate optical density, using the imageJ tool (National Institutes of Health and the Laboratory for Optical and Computational Instrumentation).

To analyze inflammation, we will use monoclonal antibodies to interleukin (IL)-17 (Mybiosource) to detect IL-17 and IL-1B polyclonal antibodies (Bioss) to detect IL-1B. As for angiogenesis, we will use CD31 antibodies (WM59; Genetex) to detect CD31 levels in tissue samples.

### Statistical Analysis

A statistical analysis will be accomplished for all clinical outcomes analyses. The data distribution will be tested by using the Kolmogorov-Smirnov test. SEs, 95% CIs, and *P* values will be reported. *P* values of ≤.05 will be considered significant for differences.

## Results

Our study is at the protocol development stage, and as such, no results are available. The experimental procedures in this study will be done after a preliminary study for assessing its feasibility and ethical approval from Animal Research Ethical Committee, Faculty of Veterinary Medicine, Institut Pertanian Bogor University (approval number: 208-2021 IPB).

## Discussion

### Study Overview

Our study will investigate the impact of PRP-DAS application on mesh-tissue integration for the implantation of PPMs in rabbit models. We hypothesized that there would be a lower inflammatory response, higher levels of angiogenesis, and collagen deposition in hypoestrogenic rabbit models treated with a PRP-DAS.

The mesh-tissue integration process will be analyzed based on inflammatory parameters (inflammatory infiltrate, IL-17, and IL-1B expression), angiogenesis (CD31 expression), and collagen deposition (Masson trichrome staining). In the proposed study, we will attempt to develop a way to promote wound healing and mesh-tissue integration in mesh-augmented surgery for POP treatment.

PRP, as a source of concentrated growth factors, has been widely applied in the field of regenerative medicine. Several studies show the substantial clinical benefits of applying PRP in periodontal regenerative therapy, burn wound healing, and mesh-augmented surgery [8-12].

### PRP Promotes Wound Healing

A study was done to investigate the effect of PRP on skin wound healing; a full-thickness skin defect model was used, and wound healing progress was analyzed at different time points after skin injury. The results showed that compared with the control group, wound closure in the PRP group was significantly accelerated, and the wounds were cleaner and exhibited much less exudation. These results were confirmed by several other studies [8-10].

### PRP Decreases Wound Inflammatory Responses

A moderate inflammatory response is helpful to normal wound healing, though any disruption in this phase results in excessive wound healing or chronic wound formation. Several studies have shown decreases in inflammatory cell infiltration in PRP groups when compared with that in non-PRP groups [8-10].

### PRP Promotes the Angiogenesis of Wound Tissue

Angiogenesis plays critical roles in effective wound healing. The results of several studies showed that the amount of neovascularization in wound tissue significantly increased in PRP groups when compared with that in control groups. These studies confirmed the positive impact of PRP on angiogenesis by measuring the expression of CD31, a marker for evaluating vascularization and angiogenesis, and vascular endothelial growth factor, a crucial growth factor for vascular endothelial cell division and angiogenesis in wound tissue. In these studies, CD31 and vascular endothelial factor levels increased, suggesting that PRP has a positive impact on angiogenesis [8-10].

### PRP Promotes Wound Contraction and Collagen Arrangement

The expression and arrangement of collagen fibers in wounds determine the quality of tissue remodeling. In several studies, the evaluation of collagen deposition via the Masson trichromatic method showed a significant increase in collagen deposition, which was accompanied by an ordered arrangement and uniform density, in PRP groups, suggesting that PRP could provide a favorable environment for further tissue remodeling [8-10].

Our study will identify the effect of a novel PRP-DAS–PPM sandwich on wound remodeling and tissue integration. The PRP will act as an anti-inflammatory and wound healing accelerator that will improve mesh-tissue integration through a nonconventional pathway. The results of our study will provide a better understanding on the underlying regulatory mechanism of PRP, especially among individuals in a hypoestrogenic state.

It is expected that there will be a lower inflammatory response, higher levels of angiogenesis, and collagen deposition in hypoestrogenic rabbit models treated with a PRP-DAS. The expected results are comparable to those of studies that were performed by Ávila et al [10] and Parizzi et al [6]. Ávila et al [10] showed that there was a significant difference in the number of inflammatory cells between the group with PRP and the group without PRP (*P*=.01) at 90 days, whereas Parizzi et al [6] showed that the group with PRP-coated meshes had a lower inflammatory infiltrate count at 30 days and exhibited increased collagen III deposition at 90 days.

The strengths of our study are the use of hypoestrogenic rabbit models and the use of the vesicovaginal space as the mesh implantation site. Similar studies used nonhypoestrogenic rabbit models and implanted meshes in the peritoneal space, which was sterile [10]. Therefore, previous studies did not describe the actual conditions for vaginally implanted meshes in menopausal women with normal vaginal flora (ie, the environment surrounding the mesh and how such an environment reduced a wound’s healing potential). Our study will also be the first to perform PRP-DAS preparation. Further, a limitation of our study was the extensive training that each operator needed to undergo prior to performing the procedure.

## Appendix

Multimedia Appendix 1 Peer-review report 1 by the Kementerian Riset Dan Teknologi (Ministry of Research and Technology) / Badan Riset Dan Inovasi Nasional (National Research and Innovation Agency) - Deputi Bidang Penguatan Riset Dan Pengembangan (Deputy for Strengthening Research and Development) (Jakarta, Indonesia).

Multimedia Appendix 2 Peer-review report 2 by the Kementerian Riset Dan Teknologi (Ministry of Research and Technology) / Badan Riset Dan Inovasi Nasional (National Research and Innovation Agency) - Deputi Bidang Penguatan Riset Dan Pengembangan (Deputy for Strengthening Research and Development) (Jakarta, Indonesia).

## Abbreviations

ARRIVE: Animal Research Reporting In Vivo Experiments
DAS: decellularized amnion scaffold
IL: interleukin
POP: pelvic organ prolapse
PPM: polypropylene mesh
PRP: platelet-rich plasma
