# Supplementary material for: Mesh-Tissue Integration of Platelet-Rich Plasma–Decellularized Amnion Scaffold–Polypropylene Mesh Sandwiches Implanted in the Vesicovaginal Spaces of Hypoestrogenic Rabbit Models: Protocol for a Randomized Controlled Trial
Source: JMIR Res Protoc. 2022 Aug 9;11(8):e37942. doi: 10.2196/37942 (PMC9399874; doi:10.2196/37942)
Supplement: Multimedia Appendix 1 [file resprot_v11i8e37942_app1.pdf]

KEMENTRIAN RISET DAN TEKNOLOGI /
BADAN RISET DAN INOVASI NASIONAL
DEPUTI BIDANG PENGUATAN RISET DAN PENGEMBANGAN
Gedung BJ Habibie Lantai 19 – 20, Jalan M.H. Thamrin Nomor 8, Jakarta 10340
Telepon: (021) 3169707; Faksimile: (021) 3101728, 3102368
Laman: www.risbang.ristekbrin.go.id

Reviewers' Comments for Author's Rebuttal

Manuscript titled "The Impact of Platelet-Rich Plasma-Decellularized Amnion Scaffold Sandwich on Polypropylene Mesh Implanted in Vesicovaginal Space of Hypoestrogenic Rabbit Model: Analysis of Mesh-Tissue Integration"

Please write down your response or revision in **"Author's Response / Revision"** column. Locate the changes you've made or the sentence that you refer to as their page and line in **"Location in Text"** column. Please also mark the changes you have made **in your manuscript** by highlighting or changing the font color.

Reviewer

No.	Aspect	Comments	Author's Response / Revision	Location in Text
	Title	**REVIEWER 1**<br>Appropriate<br><br>**REVIEWER 2**<br>The title is appropriate.	-	Title

	Abstract	**<u>REVIEWER 1</u>**<br>Need some corrections:<br>1. Please determine the type of mesh used in this study<br>**<u>REVIEWER 2</u>**<br>1. Please correct the typos in abstract.	Reviewer 1:<br>1. Added “polypropylene mesh”.<br>Reviewer 2:<br>1. The typos are revised.	Abstract<br>Page 1
	Introduction	**<u>REVIEWER 1</u>**<br>Need some corrections:<br>1. Please determine the type of mesh used in this study<br>**<u>REVIEWER 2</u>**<br>1. Please correct the typos in introduction.<br>2. The objectives are clearly described.<br>3. The manuscript clearly articulate the novelty and impact of the study.<br>4. The gap of knowledge is clearly articulated.	Reviewer 1:<br>1. Added “polypropylene mesh”.<br>Reviewer 2:<br>1. The typos are revised.	Introduction<br>Page 2.
	Methods	**<u>REVIEWER 1</u>**<br>Appropriate<br><br>**<u>REVIEWER 2</u>**<br>Appropriate.	-	Methods
	Results	**<u>REVIEWER 1</u>**<br>Appropriate<br><br>**<u>REVIEWER 2</u>**<br>Appropriate.	-	Results
	Discussion	**<u>REVIEWER 1</u>**<br>Appropriate<br><br>**<u>REVIEWER 2</u>**<br>Appropriate.	-	Discussion
