# Supplementary material for: Mesh-Tissue Integration of Platelet-Rich Plasma–Decellularized Amnion Scaffold–Polypropylene Mesh Sandwiches Implanted in the Vesicovaginal Spaces of Hypoestrogenic Rabbit Models: Protocol for a Randomized Controlled Trial
Source: JMIR Res Protoc. 2022 Aug 9;11(8):e37942. doi: 10.2196/37942 (PMC9399874; doi:10.2196/37942)
Supplement: Multimedia Appendix 2 [file resprot_v11i8e37942_app2.docx]

**Reviewers’ Comments for Author’s Rebuttal**

Manuscript titled “The Impact of Platelet-Rich Plasma-Decellularized Amnion Scaffold Sandwich on Polypropylene Mesh Implanted in Vesicovaginal Space of Hypoestrogenic Rabbit Model: Analysis of Mesh-Tissue Integration”

Please write down your response or revision in **“Author’s Response / Revision”** column. Locate the changes you’ve made or the sentence that you refer to as their page and line in **“Location in Text”** column. Please also mark the changes you have made **in your** **manuscript** by highlighting or changing the font color.

**Reviewer**

| **No.** | **Aspect** | **Comments** | **Author’s Response / Revision** | **Location in Text** |
| --- | --- | --- | --- | --- |
|  | Title | **REVIEWER 1**  Sudah sesuai  **REVIEWER 2**  Sudah sesuai | - | Title |
|  | Abstract | **REVIEWER 1**   1. Mohon cantumkan tipe mesh yang digunakan dalam penelitian ini 2. Tindakan pembedahan di bidang uroginekologi adalah secara klinis, sehingga fokus saja pada *novelty* penelitian ini karena keterbatasan waktu dan sumber daya.   **REVIEWER 2**   1. Mohon perbaiki kesalahan pengetikan. | **REVIEWER 1:**   - - - 1. Menambahkan “polypropylene mesh”.       2. Menyesuaikan dengan arahan reviewer.   **REVIEWER 2:**   - - - 1. Kesalahan pengetikan telah diperbaiki. | Abstract  Page 1 |
|  | Introduction | **REVIEWER 1**   1. Mohon cantumkan tipe mesh yang digunakan dalam penelitian ini 2. Bagaimana hasil penelitian tanpa oophorektomi terhadap luaran?   **REVIEWER 2**   1. Mohon perbaiki kesalahan pengetikan. 2. Tujuan penelitian tergambarkan degan jelas. 3. Apakah secara teori proses inflamasi, angiogenesis dan remodeling terjadi sendiri-sendiri dan apakah saling mempengaruhi? 4. Mengapa sel otot polos vagina tidak dinilai juga dalam penelitian, sedangkan pada wanita menopause terjadi juga apoptosis sel otot polos dan tujuan mesh adalah untuk memperkuat vagina? | **REVIEWER 1:**   - - - 1. Menambahkan “polypropylene mesh”.       2. Penelitian di 2016 hanya menggunakan penilaian histologis saja. Berdasarkan penelitian tersebut, penambahan PRP secara signifikan mempercepat proses penyembuhan luka. Kekurangan dari penelitian ini adalah tidak menggunakan kelinci hipoestrogenik.   Penelitian lain yang melakukan penilaian fase inflamasi menggunakan ekspresi TNF-a menunjukkan perubahan yang tidak bermakna, oleh karena itu akan digunakan biomarka lain pada penelitian ini.  **REVIEWER 2:**   - - - 1. Kesalahan pengetikan telah diperbaiki.       2. Masing-masing fase ini saling mempengaruhi, tapi tidak selalu. Oleh karena itu pengukuran dilakukan untuk masing-masing fase       3. Penelitian ini bertujuan untuk menilai biokompatibilitas jaringan dengan mesh, sehingga yang dipikirkan untuk dinilai adalah masing-masing fase penyembuhan luka. | Introduction  Page 2. |
|  | Methods | **REVIEWER 1**   1. Baik pada hewan dan manusia, oophorektomi akan menyebabkan penurunan kadar estradiol, tapi tubuh dapat tetap menghasilkan estrogen. Setelah dieuthanasia, sebaiknya periksakan lagi kadar estradiol dalam darah. 2. Kadar estrogen antar hewan bervariasi, bagaimana kalau kadar awal variasinya cukup jauh berbeda? 3. Mengapa pada penelitian ini mesh akan diletakkan pada dinding anterior dan posterior vagina, sedangkan terdapat perbedaan kekuatan antara keduanya? Apakah tidak akan menjadi perancu?   **REVIEWER 2**   1. Apakah analisis erosi hanya akan dilakukan saat euthanasia? 2. Mohon tambahkan satuan waktu pemeriksaan pada penelitian. 3. Bagaimana pengaruh perbedaan ketebalan mukosa kelinci dengan manusia terhadap luaran penelitian ini? | **REVIEWER 1**   1. Kadar estradiol akan diperiksakan juga setelah dilakukan euthanasia. 2. Oleh karena itu yang dijadikan patokan adalah penurunan kadar estradiol hingga 50% dari kadar awal. 3. Penempatan mesh pada dinding anterior dan posterior tidak berpengaruh terhadap penilaian biokompatibilitasnya. Masih diperlukan *preliminary study* untuk memastikan hal ini   **REVIEWER 2**   1. Tidak, kelinci akan dibedakan menjadi 4 kelompok dan dieuthanasia pada hari yang berbeda (hari ke-7, 14, 28 dan 90) 2. Satuan waktu sudah ditambahkan. 3. Mukosa pada manusia lebih tebal daripada kelinci, sehingga dipikirkan apabila tidak terjadi erosi pada kelinci, maka luaran yang akan ditemukan pada manusia akan lebih baik. Tentu perlu diingat bahwa banyak faktor lain yang dapat mempengaruhi hal ini, termasuk teknik operasi. | Methods |
|  | Results | **REVIEWER 1**  Sudah sesuai  **REVIEWER 2**  Sudah sesuai | - | Results |
|  | Discussion | **REVIEWER 1**   1. Mengapa diperlukan 3 komponen pemeriksaan untuk inflamasi (ININ, IL-17 dan IL-1B)? 2. Apa perbedaan antara IL-17 dan IL-1B? 3. Pada kelinci, POP diperiksa 3 bulan pasca-oophorektomi, sedangkan pada manusia POP terjadi belasan tahun setelah menopause. Apakah ini tidak akan menjadi perancu?   **REVIEWER 2**   1. Proses maturasi belum tergambarkan pada penelitian 2. Apakah terdapat standard dari volume PRP? 3. PRP jenis apa yang akan digunakan pada penelitian? | **REVIEWER 1**   1. Supaya penelitian lebih lengkap dan penilaian lebih komprehensif. 2. IL-17 diproduksi oleh neutrofil, sedangkan IL-1B dari makrofag. 3. Tidak, karena hal yang menjadi patokan adalah kadar estrogennya.   **REVIEWER 2**   1. Proses maturasi digambarkan oleh rasio kolagen tipe I dan III. 2. Sejauh ini tidak ada standar volume PRP. Hal yang perlu dikonfirmasi adalah kandungan plateletnya. 3. PRP yang akan digunakan adalah *pure* PRP (pPRP). | Discussion |
